# On Optical Detection of Densely Labeled Synapses in Neuropil and Mapping Connectivity with Combinatorially Multiplexed Fluorescent Synaptic Markers

**DOI:** 10.1371/journal.pone.0008853

**Published:** 2010-01-22

**Authors:** Yuriy Mishchenko

**Affiliations:** Department of Statistics and Center for Theoretical Neuroscience, Columbia University, New York, New York, United States of America; The Mental Health Research Institute of Victoria, Australia

## Abstract

We propose a new method for mapping neural connectivity optically, by utilizing Cre/Lox system Brainbow to tag synapses of different neurons with random mixtures of different fluorophores, such as GFP, YFP, etc., and then detecting patterns of fluorophores at different synapses using light microscopy (LM). Such patterns will immediately report the pre- and post-synaptic cells at each synaptic connection, without tracing neural projections from individual synapses to corresponding cell bodies. We simulate fluorescence from a population of densely labeled synapses in a block of hippocampal neuropil, completely reconstructed from electron microscopy data, and show that high-end LM is able to detect such patterns with over 95% accuracy. We conclude, therefore, that with the described approach neural connectivity in macroscopically large neural circuits can be mapped with great accuracy, in scalable manner, using fast optical tools, and straightforward image processing. Relying on an electron microscopy dataset, we also derive and explicitly enumerate the conditions that should be met to allow synaptic connectivity studies with high-resolution optical tools.

## Introduction

The problem of reconstructing synaptic connectivity in neural circuits has recently attracted much attention [1], [2], [3], [4], [5], and a few projects for reconstructing connectivity in different systems, such as *C. Elegans*, *Drosophila*, or mouse, had been suggested or are already under way. Since the time of Ramon y Cajal [6], neuroscientists have been intensely curious about anatomical structure of the nervous system, and much information about the large-scale connectivity of the brain had been already collected over the past century. Still, in the past decade new understanding of the role of collective behavior of many interacting neurons in information processing in brain emerged [7], [8], [9], [10], [11]. This bestowed new meaning and new importance on the old challenge of comprehensive, detailed reconstructions of large-scale neural connectivity in the brain.

Some of the recent such projects propose coarse reconstructions of neural connectivity using large scale injections of tracers or viral gene transfer [12], diffusion tensor imaging [13], [14], or sparsely expressed fluorescent markers [15]. Other projects focus on the electron microscopy (EM) for detailed reconstructions down to the level of individual synapses [1], [2]. EM is widely accepted to be the only tool for such reconstructions of neural connectivity with the precision of individual synapses. In this paradigm, the process of reconstruction is approached in the following way: tiny synaptic contacts are first located in neuropil using EM; pre-synaptic axons and post-synaptic dendrites are identified in EM images for each synaptic contact; axonal and dendritic projections are traced to their respective cell bodies using EM images over macroscopically large distances (e.g., see [16]). Unfortunately, this paradigm suffers from two major drawbacks – the acquisition rate of the electron microscopy data is extremely low, and tracing of the neural projections in EM images through densely packed neuropil has proven to be very difficult [2], [17], [18], [19], [20]. Such reconstructions are also vulnerable to imaging and analysis errors, where every error in a long sequential trace of an axon can lead to devastating consequences for the entire reconstruction by causing large number of that axon's synapses, downstream of the site of error, to be lost or mislabeled. Expected frequency of such errors, unfortunately, is quite high [17].

Recently, an original light microscopy (LM) alternative to difficult EM reconstructions had been proposed [3], [21]. In this approach, termed Brainbow, neurons are made express random mixtures of fluorophores with different emission wavelengths (e.g. nGFP, YFP, etc.), thus, labeling somas, axons and dendrites of different neurons with a variety of different colors. Brainbow allows one to significantly reduce the difficulty associated with the tracing of neural projections, because axons and dendrites of every neuron have the same color and, thus, can be traced more easily. In particular, using Brainbow mice [3], [21], J. Lichtman and colleagues were able to complete reconstructions of a number of larger axons and their synapses in several neural circuits in mice [21], [22], [23].

Unfortunately, Brainbow technique is only helpful when the target population of neurons is sparse. Because neurites are packed so densely in neuropil (∼10–40 neurites per voxel of a typical diffraction limited microscope), for a densely labeled population of neurons the fluorescence from all such neurites tends to blend together, making individual neurites indiscernible. If it was possible to modify Brainbow to label only synaptic regions of neurons, as opposed to entire bodies, this problem of dense packing could be circumvented because synapses in neuropil are “sparse”, 1–2 synapse per µm^3^. Perhaps even more significantly, such system would allow mapping neural connectivity in a dramatically simpler way, without tracing of individual neural projections. Assuming that synapses of different neurons could be tagged with distinct mixtures of fluorophores using the Brainbow construct, the fluorescence color of different synapses could be used to immediately identify the pre- and post-synaptic cells at each synaptic connection. This would allow mapping neural connectivity using optical tools, rapidly, in scalable manner, and without tracing neural projections.

Unfortunately, it is widely believed in the neuroscience community that LM cannot be used to successfully observe individual synapses, due to resolution limitations, and EM is the only tool capable of that. However, with the advent of LM super-resolution techniques [24], [25], [26], [27], [28], it now becomes possible to study individual synapses optically. E.g., [28] reports observation of individual synaptic puncta already with a diffraction limited LM used with a ultra-thin-slices preparation. It is still unclear, however, what the minimal conditions should be for such optical observation of synapses, or how accurately the composition of the fluorophore mixtures at different individual synapses can be determined.

In this paper, we use a 130 µm^3^ block of juvenile rat hippocampal neuropil [29], completely reconstructed from a stack of high resolution electron micrographs, to address these questions. We simulate LM observation of the population of synapses in that EM data, and show that Structured Illumination Microscopy (SIM) [26] and Isotropic Diffraction Limited Microscopy (IDLM) [28] could be used to observe these synapses successfully. Moreover, we find that the identity of the fluorophores expressed at each synapse could be determined with the reliability of up to 99%, using these tools. Fluorophores may be simply tagged to the pre- and post-synaptic sides of the synaptic clefts, e.g., using Munc-13 or PSD-95; no chemical binding across synaptic clefts, as in [30], is necessary. While one might think that random oppositions of the fluorophores from nearby neurites could pose a problem for such an approach, our analysis using EM data shows that such false oppositions would be extremely rare with SIM or IDLM.

Our results have important implications for studies of synaptic connectivity using optical tools. In particular, we show that by expressing a pre-synaptic marker in one population of neurons and a post-synaptic marker in another population of neurons, and then collecting thus labeled synaptic puncta with the methods described below, the connectivity between different classes of neurons can be reliably measured over macroscopically large regions of brain using optical tools. Furthermore, using *N_c_*≈10–20 spectrally distinct fluorescent markers multiplexed on synapses, it is possible to efficiently map neural circuits composed of ∼100–1000 neural classes simultaneously. Reconstructions of even bigger circuits are possible by combining such measurements from different animals, e.g., using the method of [31]. In this way, the reconstruction of the connectivity in the entire *Drosophila* brain can be accomplished using *N_c_* = 10–20 spectrally distinct fluorescent synaptic marker and imaging of ∼100–1000 animals.

## Materials and Methods

### 2.1. Preparation of the Electron Microscopy Data

The EM data used in this work comprises a stack of 93 electron micrographs of a block of hippocampal neuropil, available publically from Synapse Web (synapses.clm.utexas.edu, volume P21AA). Briefly, this volume was prepared from a hippocampal slice of P21 male rat of the Long-Evans strain, fixed via perfusion through the heart with glutaraldehyde fixative, and then processed with potassium ferrocyanide-reduced osmium, osmium, and aqueous uranyl acetate. Ultrathin 50 nm sections were cut from the middle of the slice, 120–150 µm from the air surface in stratum radiatum, at a distance of 200 µm from the CA1 cell body layer. Sections were photographed using EM, and aligned into a 3D volume using Reconstruct software. For further details of the tissue preparation and imaging the reader is referred to the relevant publication [29].

**Figure 1:**
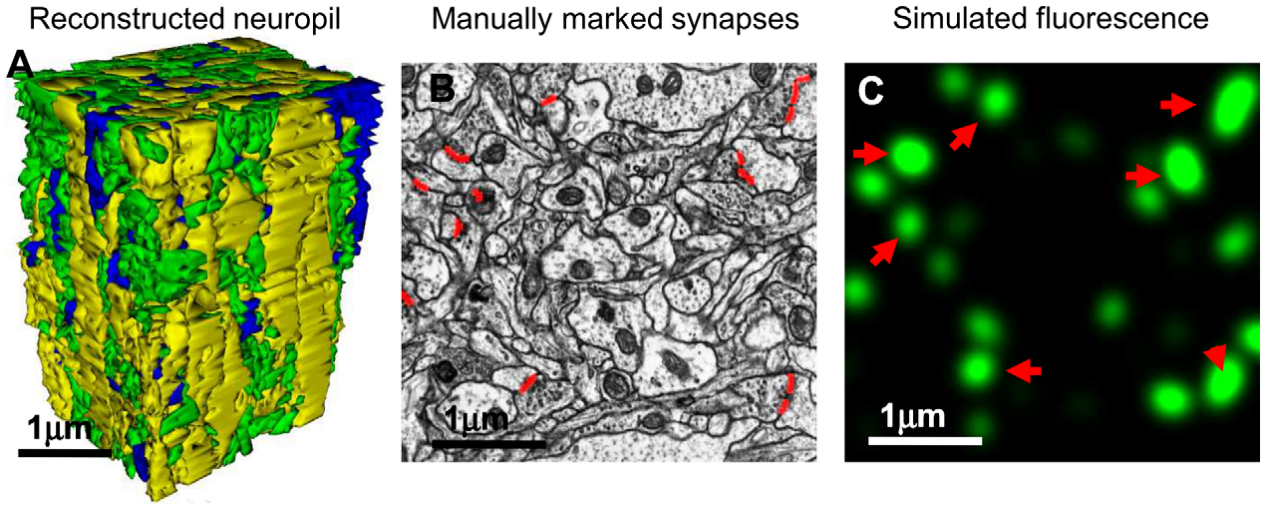
Electron Microscopy reconstruction data. A) Using a 130 µm^3^ block of hippocampal neuropil imaged with high-resolution electron microscopy, we investigate the possibility of detecting individual synapses optically. This block was fully segmented by the author, and all synapses were explicitly labeled. For illustration purposes, here is shown the 3D model of the reconstruction of all neuronal processes in said block, colored according to process type – yellow for dendrites, green for axons, and blue for glial processes. B) An example of the manual markup of the synapses within one electron micrograph (red lines). C) Simulated fluorescence from marked synapses (here, for isotropic diffraction limited microscopy, IDLM). Red arrows indicate synapses located directly inside shown EM section (B).

Sub-volume of this dataset, used for analysis here, measured 4.5×6.7×4.5 µm^3^ at the resolution of 8×8×50 nm/pixel. This volume was fully segmented into the constituent axons, dendrites, and glia, using the automated segmentation approach of [17], and all synapses in the volume were consequently manually labeled, Figure 1. The volume was found to contain fragments of 30 dendrites and 256 axons. 250 synapses were found, corresponding to the synaptic density of 1.85 µm^−3^. Matlab's proofreading GUI, developed for the automated segmentation approach of [17], was used to mark up synapses: each synapse was marked on the computer with a distinct color along its entire span using this tool, and then a single-pixel line representation was produced for each synapse, where each pixel was viewed as a 8×50 nm “vertical” slab representing the surface of the synapse. Additional adjustment of all synaptic areas was performed in order to correct for that obliquely running synaptic surfaces were reduced to such vertical slabs. I.e., a synapse that ran obliquely to the plane of the electron micrographs, e.g., at an angle of 45 degrees, was represented with a 50 nm vertical slab, even though the actual length of its cross-section was 50 nm/cos(45°)≈70 nm. It may be shown by a straightforward calculation that on average this effect leads to under-representation of the synaptic areas by a factor of π/2.

Fluorescence was simulated by convolving that map of labeled synapses with the point spread function of a particular light microscope, modeled as a Gaussian with the lateral dimensions *d*_xy_ and the axial dimension *d*_z_ (Figure 1C).

### 2.2. Evaluating the Fraction of Synapses That Can Be Explicitly Resolved with LM

To determine how many synapses could be explicitly resolved with a given light microscope (i.e., isolated into separate puncta), we thresholded the simulated fluorescence field, *I*(*x*,*y*,*z*), Figure 1C, at various levels of intensity from 0 to max[*I*(*x*,*y*,*z*)], and then found all separate fluorescent puncta by constructing distinct supra-threshold regions contiguous in three-dimensional 26-connected topology, using Matlab. A synapse was said to be resolved if a punctum could be found that spatially covered it exclusively for at least one threshold. We then counted the fraction of all resolved synapses for different imaging conditions.

### 2.3. Threshold Method for Detecting Synapses with LM

**Figure 2:**
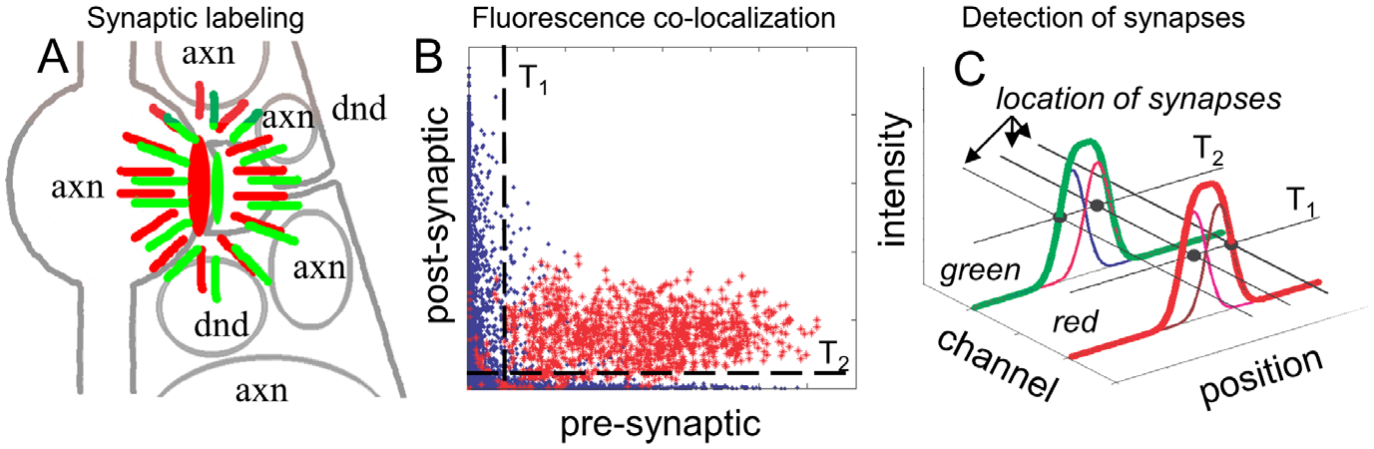
Schematic explanation of synapse detection using co-localization of fluorescence from different pre- and post-synaptic markers. A) Schematic diagram of the synaptic Brainbow, with a red fluorophore on the pre-synaptic side and a green fluorophore on the post-synaptic side of a synaptic cleft. Spatial correlation of the fluorescence from the pre- and post-synaptic fluorophores, occurring due to their proximity across synaptic cleft, allows detecting synapses optically without explicitly resolving them. Due to absence of the fluorophores in the bulk of the axonal and dendritic cytoplasm, nearby processes do not interfere with the detection process even when all neurons are labeled, unlike in regular Brainbow. B) Due to close spatial co-localization of the pre- and post-synaptic fluorophores across the synaptic cleft, their fluorescence intensity is closely correlated near labeled synapses. In this figure we show a simulated scatter plot of the fluorescence intensity in IDLM. Blue dots represent voxels far away from one labeled synapse (further than ≈200 nm), and red dots represent voxels closer than ≈200 nm. One can threshold this diagram with certain thresholds, T_1_ for the pre-synaptic marker and T_2_ for the post-synaptic marker (dashed lines), in order to separate the proximal (red) from distant (blue) voxels, and thus detect presence of a synapse. C) Using correlations in the fluorescence from the pre- and post-synaptic markers, synapses may be detected even when they cannot be explicitly resolved into isolated puncta. Illustrated here are three “synapses”, fluorescence from which individually is shown with thin blue, magenta and brown lines. These are observed using two fluorescent markers, green and red. First synapse is tagged only with “green” marker, second synapse is tagged with “green” and “red” markers, and third synapse is tagged with “red” marker. Combined fluorescence from these synapses is shown with thick red and green lines, for the two markers respectively. Even though none of synapses can be seen separately in either green or red channels, by thresholding fluorescence with appropriate thresholds, T_1_ and T_2_, three different supra-threshold fluorescence patterns (black dots) indicate presence of three synapses.

While one can detect synapses with LM by looking for explicitly isolated fluorescent puncta, one can also use a more powerful, yet simpler, prescription for detecting synapses *implicitly*. Specifically, consider a synapse labeled with two fluorophores, a fluorophore AFP on the pre-synaptic side and a fluorophore BFP on the post-synaptic side. Because of the spatial proximity of these two fluorophores across the synaptic cleft (i.e., ∼10–50 nm apart), the fluorescence from these fluorophores will be tightly correlated in the region near labeled synapse, Figure 2A and 2B. This correlation may be quantified and used to detect the synapse even when it cannot be resolved as an isolated punctum, Figure 2C.

A variety of prescriptions for detecting such correlation may be suggested. Here, we analyze a simple algorithm, where we say that a synapse with a pre-synaptic fluorophore AFP and a post-synaptic fluorophore BFP is present if a voxel can be found in LM images where the fluorescence intensity from AFP and BFP is *simultaneously* above a predefined threshold. More concretely, for each voxel we test whether the fluorescence from a specific fluorophore is above certain threshold *T_i_*. For each voxel, thus, we assign a pattern of all fluorophores that are “supra-threshold” there. For each pattern, thus, we count the total number of associated supra-threshold voxels. If such count is above certain second threshold *T_v_*, we say that a synapse tagged with that pattern of fluorophores is present.

### 2.4. Evaluating the Fraction of Synapses That Can Be Detected with the Threshold Method

To determine how many synapses could be detected using the threshold method, fluorescence field simulated from the EM dataset, *I*(*x*,*y*,*z*), was first down-sampled to “optical” voxels. If the original EM voxel had the size of 8×8×50 nm, the optical voxel was chosen to have the size equal to 1/4 of the light microscope's resolution. E.g., for IDLM resolution of *d*_xy_ = *d*_z_ = 200 nm the optical voxel had dimensions of 50×50×50 nm. For each optical voxel, the mean and the variance of the count of detected photons were computed. Using these counts, we calculated how many synapses could be identified with the threshold method, and compared that with the EM data.

Specifically, we inspected a set of 100 choices for *T*_i_, spanning the full range of fluorescence intensity from 0 to max[*I*(*x*,*y*,*z*)], and found the choice of *T_i_* that produced the lowest total number of errors. For the sake of reducing the computational burden, we pre-computed and pre-ordered the individual fluorescence contributions from all synapses for each voxel. Then, for different thresholds *T*_i_, we found the number of synapses contributing supra-threshold at each voxel. If two or more synapses contributed supra-threshold at certain voxel, an error was recorded regarded as the detection of a false pattern blending two top synapses together. E.g., if one of the supra-threshold synapses had a fluorophore AFP, and the other had a fluorophore BFP, such voxel would be identified by the threshold method as a “false” synapse tagged with AFP and BFP together, even though no such synapse existed in reality. If only one synapse contributed supra-threshold at a voxel, that synapse was said to be detected correctly. If, for a given synapse, no voxels could be found where that synapse was supra-threshold, such synapse was said to be lost.

Fluorescence at each voxel, *I*(*x*,*y*,*z*), was computed as follows. The photons arrive to voxels from nearby fluorophore molecules in a random Poisson process; likewise, the fluorophore marker molecules bind to the nearby synapses in a random Poisson process. The count of photons at different voxels, therefore, is described by a random double-Poisson process. For analytical tractability, we model here the above two Poisson processes using Normal distributions with scaled variance. Specifically, the number of the fluorophore molecules at a synaptic surface at location 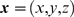 is described by a normal distribution with the mean 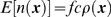 and the variance 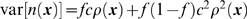,
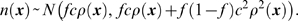
(1)Here, 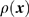 is the density of the synaptic material at location 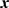, in µm^2^/pixel, *c* is the average concentration of the fluorophore molecules on the synaptic surface, in µm^−2^, and *f* = 0.5 is the fraction of the neurons that express one fluorophore in Brainbow settings. 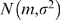 stands for the Normal random variable with the mean *m* and the variance *σ* ^2^. Bold notation refers to the vectors; i.e., for a point in a three-dimensional space with the coordinates 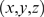 we write simply 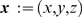. The variance in (1) consists from two terms: the Poisson variance in the number of the fluorophore molecules bound at the synaptic surface at location 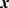, and the variance in the amount of the synaptic material at 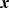 due to random expression in Brainbow.

The number of photons arrived at voxel 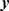 from location 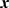 is described by
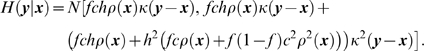
(2)Here, 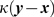 is the kernel corresponding to the microscope's point spread function, and *h* is the “photon budget” parameter, i.e., the average number of photons received in the detector per one emitting fluorophore molecule. The variance is composed from several terms, including the pure Poisson variance in the photon counts, 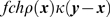, and the variance carried over and amplified by *h* from 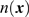. The final photon count at voxel 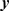, and its variance, is produced by summing Eq. (2) over all 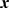, assuming that the photon emission processes at different locations 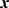 are independent.

## Results

### 3.1. Theoretical Bounds for Detecting Synapses with LM

**Figure 3:**
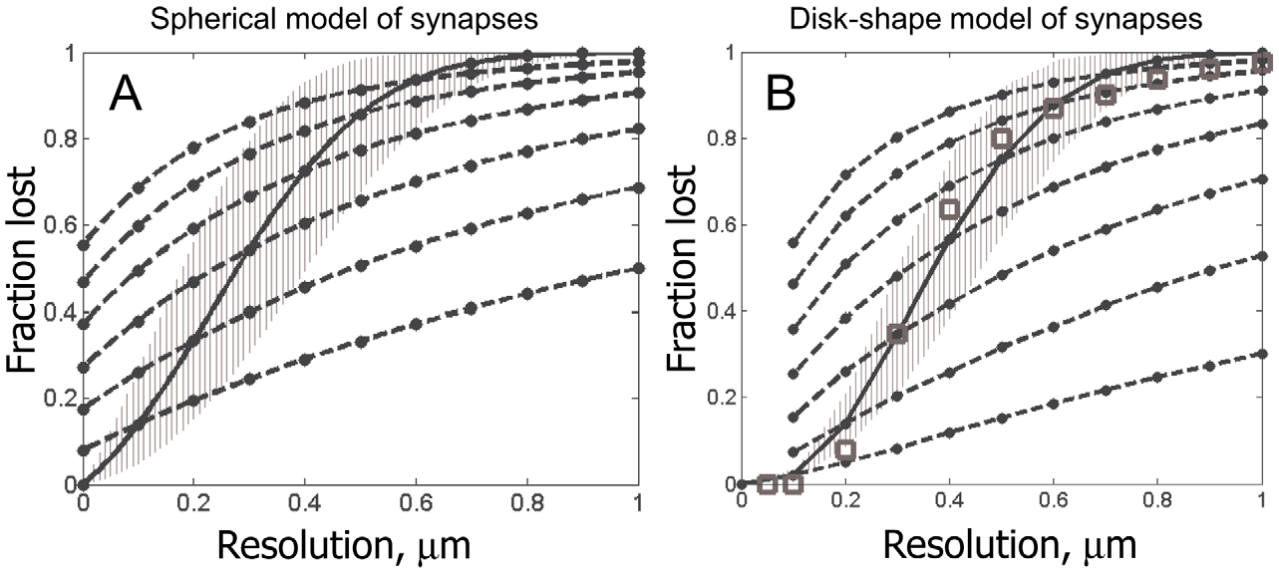
Theoretical estimation of the fraction of directly optically resolved synapses for different LM resolutions. A) The fraction of unresolved synapses in the model of “spherical” synapses. Solid black line is for isotropic LM resolution; and the range corresponding to different synaptic densities, from 1 µm^−3^ to 2 µm^−3^, is also shown (grayed area). Dashed lines are for LM instruments with anisotropic resolution, in which case the X-axis specifies the *axial* resolution of the instrument. Legend in A is also for B. B) The fraction of unresolved synapses in the model of “disk-shaped” synapses. Also shown is the fraction of optically resolved synapses determined directly from our EM reconstruction (squares).

We begin this section with a simple calculation involving several basic facts known for mammalian neuropil from neuroanatomy: a) distribution of synapses in neuropil is consistent with a uniform random distribution with the mean density *ρ* = 1–2 µm^−3^ (except maybe at small distances of the order of the synapse size) [32], [33], and b) synapses in mammalian neuropil can be viewed as small disk-shaped objects *q* = 150–300 nm in diameter [34], [35], [36], [37]. Then, consider the problem of detecting two synapses with a light microscope with resolution *d*. For simplicity, we first neglect the disk-shape of synapses. Then, two synapses can be resolved if and only if the distance between their centers, *D*, is greater than 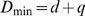. For uniformly distributed synapses, the probability that two synapses will be in such a configuration can be calculated,
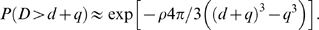
(3)If the resolution is anisotropic, *d*_xy_ laterally and *d*_z_ axially, this formula can be modified,
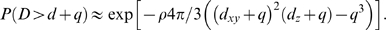
(4)In Figure 3A, we plot 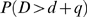 for different values of *d_xy_* and *d_z_*. For a good confocal microscope, the most widely used instrument in the neuroscience community, the best lateral resolution that can be achieved is *d_xy_*≈0.2 µm and *d_z_*≈0.6 µm. As can be seen in Figure 3A, for such a microscope the probability of blending two nearby synapses is over 50%. Likewise, from Figure 3A we see that the probability of seeing an isolated synaptic punctum becomes extremely small for resolutions worse than 1 µm (i.e., one loses detection of all synapses). Yet, we also see that the simplest super-resolution technique such as Structured Illumination Microscopy (SIM), *d_xy_*≈*d_z_*≈0.1 µm [26], may be able to successfully resolve at least 90% of all synapses.

We now try to include the disk-shape of synapses in our model calculation. The probability that two disk-shaped synapses can be resolved is given by the formula,
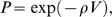
(5)where the excluded volume *V* is calculated in the following way,
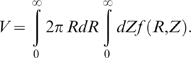
(6)Eq. (6) is analogous to Eq. (4), except that we re-write the excluded volume 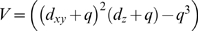 as an integral over the lateral and the axial dimensions, *R* and *Z*, and introduce a function 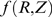 that describes the fraction of the synapses of non-spherical shape that cannot be resolved at the relative position (*R*,*Z*) (i.e. that have orientations such that their optical images blend together).

**Figure 4:**
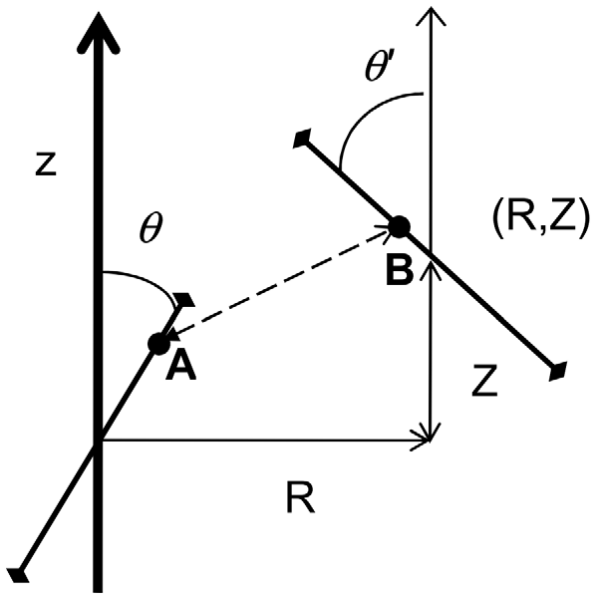
Geometry setup for calculating the fraction of unresolved synapses at a relative position (*R*,*Z*). *R* is the distance between centers of two synapses in the microscope's focal plane (lateral distance), and *Z* is the distance along the optical axis (axial distance). *θ* and *θ′* are the orientations of two synapses relative to the microscope's optical axis. Two synapses are said to not be resolved if there are any two points on their surfaces, **A** and **B**, that are closer together than the microscope's resolution limit. This condition can be expressed as a quadratic program, which should be solved numerically for each (*R*,*Z*,*θ*,*θ′*).

Computation of 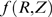 in general is very complex. To simplify this calculation here, we consider a simple geometrical model described in Figure 4. In this model, synapses are represented with line segments that can rotate in a single plane. Assuming that orientations of different synapses are isotropic, 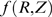 can be calculated as follows,
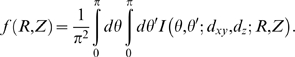
(7)

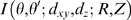
 here is the indicator function: 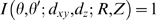 if two synapses at orientations 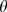 and 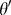 and relative position (*R*,*Z*) cannot be resolved, and zero otherwise. We integrate 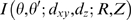 over all possible orientations 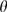 and 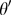 to arrive at the desired fraction, 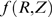.

Two synapses cannot be resolved if there are any two points on their surfaces that are closer together than the microscope's resolution limit. Thus, 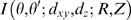 can be calculated as
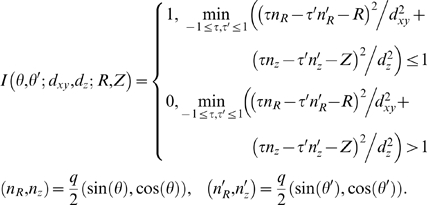
(8)In Eq.(8), *τ* and *τ′* represent the positions of some two points on the synapses, A and B in Figure 4, and the “min” statement directly corresponds to the resolvability condition above. Eq. (8) defines a so called quadratic program, and cannot be solved analytically. It can be solved numerically, e.g., using *quadprog* function provided with the computational system Matlab. Then, eq. (5–7) can be calculated numerically from the solution of (8).

Results of this involved calculation are shown in Figure 3B. We observe that elongated shape of synapses generally helps their observation: i.e., when synapses are “parallel” they look “further apart”. In particular, disk-shaped synapses are resolved well already at the regular diffraction limit (i.e., isotropic resolution of *d_xy_*≈*d_z_*≈0.2 µm), while SIM is able to resolve nearly 100% of all synapses.

These theoretical bounds match very well with the fraction of the resolved synapses calculated directly from the fluorescence simulations using the EM data below. Therefore, this strongly suggests that both IDLM and SIM can be used to resolve individual synapses with exceedingly good quality.

### 3.2. Detecting Synapses with LM: An Analysis of the Sources of Errors

**Figure 5:**
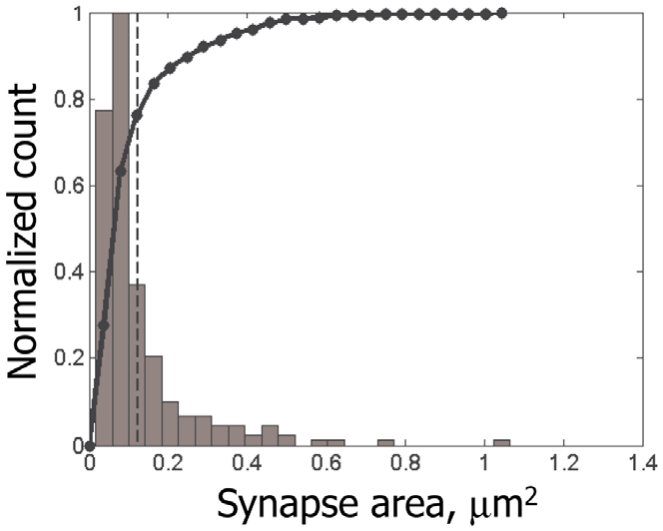
Distribution of synapse sizes measured from our EM reconstruction. Normalized histogram (gray) and respective cumulative distribution function (black line) are shown. The mean size is shown with dashed line. As can be seen here, distribution of synapse sizes is similar to the exponential distribution, with the majority of synapses measuring only up to 0.05 µm^2^.

In this section, we qualitatively understand the sources of errors in detection of synapses using fluorescent LM data. To detect a synapse tagged with a set of fluorophores (AFP, BFP, …), one needs to conclude that the fluorescence from the tags AFP, BFP, etc., is simultaneously high at some location. The fluorescence intensity is determined by two factors: the number of the fluorophore molecules bound at the target synapse, and any additional background contributions from the same fluorophore molecules bound at the nearby synapses,
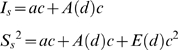
(9)Here *a* is the area of the target synapse, *c* is the concentration of the fluorophore molecules on the synaptic cleft, *A*(*d*) is the mean area of the nearby synapses within the microscope's resolution limit *d*, and *E*(*d*) is the variance in that area assuming fluorophores are expressed via a stochastic mechanism such as Brainbow. The variance *S_s_* is determined by three contributions: the Poisson fluctuations in the number of the fluorophore molecules bound at the target synapse, *ac*; the Poisson fluctuations in the number of the fluorophore molecules bound at the nearby synapses, *A*(*d*)*c*; and the variance in *A*(*d*) due to random expression in Brainbow, *E*(*d*)*c* ^2^. (Here, for clarity, we assume that the fluorescence intensity is sufficiently high, so that the shot noise in the photon counts can be neglected.) The best error rate with which a given fluorophore can be detected at the target synapse, therefore, depends on the magnitude of the change in the fluorescence signal when the fluorophore is present, 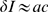, relative to the noise, *S_s_*,
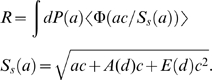
(10)The error rate, *R*, here is defined as the average total number of the false negative (i.e., existing fluorophore was not detected) and false positive (i.e., non-existing fluorophore was detected) errors per one true synapse. I.e., *R* quantifies the total number of false patterns, e.g., such that have a certain fluorophore missing or falsely included, detected per each existing synapse in a volume of neuropil. *P*(*a*) is the cumulative distribution function for the synapse sizes (black line in Figure 5), and 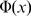 is the two-tail Normal error function. The average 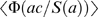 is over all synapses of the same size *a* (i.e., over *A*(*d*) and *E*(*d*)).

From Eq.(12) we see, first, that the probability of correctly detecting a fluorophore at a synapse is determined primarily by synapse's size. For larger synapses,
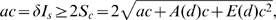
(11)the probability of making an error is very small. At the same time, fluorescence from smaller synapses is more likely to be lost on the backdrop of the background fluctuations, or detected falsely due to those fluctuations. Our first corollary, therefore, is that the majority of mistakes in *R* are from the smaller size synapses.

**Figure 6:**
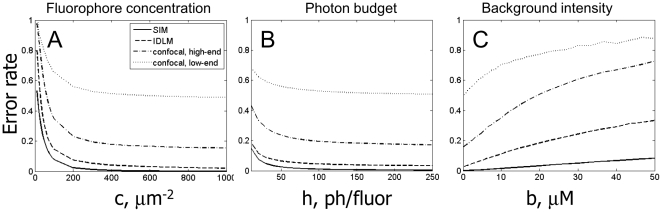
Best quality of synapse detection using the threshold method, for different imaging conditions. A) Error rate for synapse detection as the function of the fluorophore molecules concentration on the synaptic membrane. Shown are Structured Illumination Microscopy (SIM, solid line), diffraction-limited microscopy on 100 nm slices (IDLM, dashed line), high-end confocal microscopy (*d*_xy_ = 0.2 µm and *d*_z_ = 0.6 µm, dash-dotted line), and low-end confocal microscopy (*d*_xy_ = 0.4 µm and *d*_z_ = 1.25 µm, dotted line). The error rate decays towards the resolution-set limits at about 200–400 µm^−2^. Legend in A is also for B and C. B) Error rate for synapse detection as the function of the photon budget. The error rate decays towards the resolution-set limits at about 50–100 photons/fluorophore molecule. C) Error rate for synapse detection as the function of the background fluorophore pollution. The error rate monotonically grows with the background density. Although the impact of the background is substantially different for different instruments, a generally acceptable range is 1–10 µM.

For lower a*c*, the error rate in Eq. (10) is dominated by the Poisson fluctuations in the number of the fluorophore molecules bound at the synaptic surface, and can be characterized by the SNR∼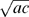. In particular, most experimentally feasible regimes are described by this case; i.e., for *c*≈1000 µm^−2^ [34], [35], [37] and a≈0.05–0.1 µm^2^ [34], [35], [36], [38] *ac*∼50–100 fluorophore molecules per a typical synapse, and the SNR is ∼7–10. Second important corollary is that, when we want to detect a smaller change in the fluorescence signal, e.g., if we want to measure the fluorophore expression level out of K possible gradations, 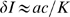, the error rate degrades as if we had a lower concentration *c_eff_* ≈ 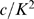. This situation is important when different neurons can produce different expression levels of the fluorescent tags, and we want to use measurements of that expression levels to additionally discriminate between neurons (rather than only use the patterns of expressed fluorophores). The above quadratic scaling, unfortunately, restricts such measurements severely; e.g., the best error rate for measuring expression level of single fluorophore with *K* = 3 gradations, using SIM and IDLM, and assuming *c_max_*≈1000 µm^−2^, is *R*≈10–20%, that can be found from Figure 6A and *c_eff_* ≈100 µm^−2^.

For resolution *d* larger than the mean inter-synaptic distance ∼1 µm, Eq.(10) is dominated by the fluorescence from the nearby synapses, 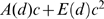. Under these conditions, synapses become impossible to detect because their fluorescence blends together. Only the largest synapses can be distinguished in that case, and the error rate can be characterized by the limiting SNR∼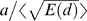.

### 3.3. Detecting Synapses with LM: An Empirical Study

In this section, we probe the process of detection of synapses using fluorescent LM data in greater detail. We use a stack of electron micrographs from a hippocampal slice of P21 male rat of the Long-Evans strain [29], available from Synapse Web (synapses.clm.utexas.edu), to directly simulate fluorescence from a dense population of fluorescently labeled synapses in that neuropil volume, and compare the fraction of synapses that can be detected there optically with the gold standard of EM.

First, we explicitly study how many synapses could be resolved into individual puncta with different LM instruments. Using the EM data above, we find that such fractions of resolved synapses are in excellent agreement with the simple theoretical calculations performed in Section 3.1. (Figure 3B).

Second, we note that, if multiple fluorophores label synaptic clefts, presence of a synapse may be often inferred even when the synapse itself cannot be resolved into an explicitly isolated punctum, Figure 2. Such implicit detection is based on observing the correlation between the fluorescence from the pre-synaptic and post-synaptic fluorophores, arising because of their extreme spatial proximity across the synaptic cleft, ∼10–50 nm. Because of spatial proximity of such fluorophores, their fluorescence will be tightly correlated in the region near the tagged synapse, Figure 2A and 2B. This correlation may be quantified and used to detect synapses even when they cannot be explicitly resolved from their neighbors, Figure 2C. One simple algorithm for such implicit detection is to record a synapse each time the fluorescence from a pair of pre- and post-synaptic fluorophores is observed to be simultaneously (i.e., at the same voxel) above a pre-defined threshold (see Methods for more details).

To test this implicit method for detecting synapses, we construct a detailed simulation of this process, where we incorporate various experimental factors such as the actual distribution of synapse sizes, *a* [µm^2^], feasible concentrations of the fluorophores on the synaptic clefts, *c* [µm^−2^], plausible photon counts per fluorophore molecule (photon budget), *h* [photons/fluorophore molecule], and the background pollution modeled as a diffuse uniform distribution of the fluorophore molecules, at volume density *b* [µM] unassociated with the labeled synapses. We consider a scenario where the fluorophores bind directly to the synaptic cleft on the pre- and post-synaptic sides (e.g., using Munc-13 and PSD-95 antibodies). Given that the spacing between the pre- and post-synaptic surfaces of the synapses in our data was much smaller than the simulated resolution (∼10–20 nm and ∼100–200 nm, respectively), we neglect the thickness of the synaptic clefts, so that both the pre- and post-synaptic fluorophores are assumed to localize on the same surface, drawn in the center of the post-synaptic density visible in the EM data. Expression of the fluorophores in different neurons is assumed to be random at probability *f* = 0.5, as in Brainbow. Each fluorophore is assumed to be present only either in axons or dendrites, and never both together. Fluorescence for each particular labeling is then simulated as described in Section 2.4.

Since the number of parameters in this simulation is very large, we explore various possible experimental regimes by choosing a single “reasonable” operating point, *f* = 0.5, *c* = 750 µm^−2^, *b* = 0.1 µM, *h* = 1000 photons/fluorophore, and a set of four LM instruments, and then investigate how quality of the synapse detection changes when one parameter is varied at a time. Quality of the synapse detection is quantified by the rate of errors per one existing synapse. E.g., if a 10×10×10 µm cube of neuropil contains ∼1000 synapses, and we are able to detect and successfully identify the patterns of expressed fluorophores on 900 out of 1000 synapses, we say that the rate of false-negative errors (lost synapses) is 100/1000 = 0.10, or 10%. If we also detect 50 patterns that do not really exist, we say that the rate of false-positive errors (falsely “found” synapses) is 50/1000 = 0.05, or 5%. The total error rate reported will be 0.1+0.05 = 0.15, or 15%.

We simulate fluorescence from the arrays of markers multiplexed on synaptic clefts, and determine how well presence of each marker on the respective synapses can be established, as described in Methods. In Figure 6, we catalogue these error rates for single markers, understanding that the error rate for a complete array can be calculated as follows. If there are *N_c_* different markers in an array, the pattern of the labels on a synapse would be determined incorrectly whenever a mistake is made in any one of its constituents. I.e., the probability to identify incorrectly a pattern of *N_c_* markers approximately is *N_c_* times higher than that for a single marker. The error rate for an array, then, approximately can be computed as *N_c_* times the error rate for a single marker, Figure 6. For a more accurate estimation, however, the dependence of the error probability on the synapse size (see Section 3.2.) should be properly taken into account for a specific choice of *N_c_* and other parameters. Such calculation can be conducted for a specific choice of the operating regime using the analytical methods described in this paper.

From our simulation, we observe that the quality of synapse detection improves monotonically for lower *f*, lower *b*, higher *c*, higher *h*, and better resolution, as should be expected. Necessary minimal fluorophore concentration appears to be *c_min_* ≈200–400 µm^−2^ (Figure 6A), and necessary photon budget *h_min_* ≈50–100 photons/fluorophore (Figure 6B). Largest acceptable fluorophore background appears to be *b_max_* ≈1–10 µM (Figure 6C). All of these figures are well within known experimental bounds: for PSD-95 the number of copies per average post-synaptic density of 360 nm in diameter was estimated to be ≈300–700 [34], [35], which corresponds to PSD-95 concentration of ≈3000–7000 µm^−2^. Similarly, [37] indicates that the densities of the synaptic proteins in post-synaptic densities are ≈3000 µm^−2^. Even with the antibody efficiencies around 30%, required fluorophore concentrations can be easily achieved. Likewise, photon counts of 10^3^–10^4^ per GFP molecule before bleaching are common [39], [40], and the background fluorophore concentrations below 1 µM are routinely achieved in practice.

The resolution-related bounds are found to be as follows: usual high-end confocal microscopy may be used if a substantial number of errors can be tolerated, the error rate ≈20–30%, while IDLM and SIM can achieve error rates below 1–5%. Between these two the difference is minor. Microscopes with the resolution worse than 1–2 µm may not be used without making the population of labeled synapses very sparse. The label sparseness, *f*, should be such that the mean distance between labeled synapses is larger than the microscope's worst resolution. Simple estimates indicate that the expression frequency for that should be below *f*≈0.001–0.01, as is also confirmed by a direct simulation (not shown).

**Figure 7:**
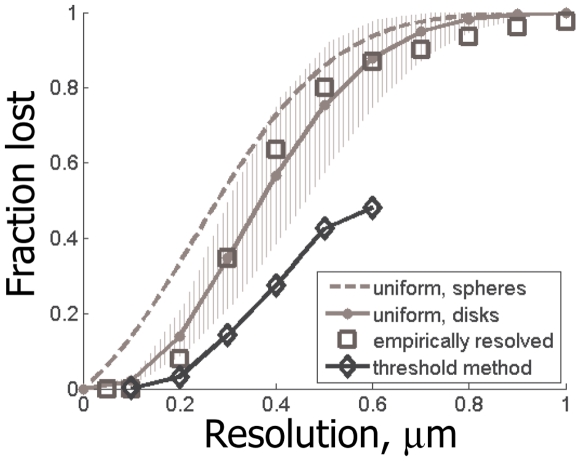
Best quality of synapse detection using the threshold method, for different LM resolutions. For comparison also shown: the fraction of optically resolvable synapses in theoretical model of “spherical” synapses (dashed gray line), the fraction (solid gray line) and the range (grayed area, for different synaptic densities from 1 µm^−3^ to 2 µm^−3^) of optically resolvable synapses in theoretical model with “disk-shaped” synapses, and the empirical fraction of optically resolvable synapses determined from our EM reconstruction data (squares).

Figure 7 summarizes the quality of the implicit synapse detection for different LM instruments. For Figure 7, we perform the simulations as described above, where we assume very large values for the parameters such as fluorophore concentration, *c*, or photon budget, *h*, thus removing from consideration all factors except for the instrument's resolution. As can be seen in Figure 7, the implicit method allows detecting synapses with substantially better quality than a naïve method based on explicit search for isolated synaptic puncta – up to 50% better. Nearly zero error rates are achieved at the resolution of 0.2 µm, with very little improvement for the instruments with yet higher power.

### 3.4. Synaptic Brainbow

Above, we establish that IDLM and SIM can be successful in detecting individual synapses in densely labeled neuropil, and determining the patterns of the fluorophores expressed on their surfaces. Based on this, we propose that a strategy for mapping the connectivity in a neural circuit will be successful, where synapses are labeled with arrays of spectrally distinct synaptic fluorophores, expressed in different combinations in different neurons via Cre/Lox system Brainbow [3], [21]. Synapses thus labeled can be found using LM, as described above, and the patterns of the fluorophores expressed on their pre- and post-synaptic surfaces can be identified. Assuming that different neurons express distinct combinations of the pre- and post-synaptic fluorophores, such patterns will immediately report the identity of the cells involved in each synaptic connection. The somas of the neurons associated with each pattern can be determined, e.g., by co-expressing same color fluorophores in the neural nuclei (see Discussion for more details). Importantly, no axons or dendrites need to be traced from synapses toward cell somas, because information about identity of every connection is available locally, at the location of every synapse. Although in Brainbow only 50% of cells express each particular fluorophore, by multiplexing many fluorophores combinatorially on synaptic clefts, nearly 100% coverage may be achieved: every synapse will be labeled with at least one fluorophore, and so can be observed. It is also possible to express different fluorophores in different cells not stochastically, but using defined genetic promoters, e.g., as in UAS/Gal4 libraries.

## Discussion

By using a 130 µm^3^ block of serial electron micrographs [29], where we explicitly reconstructed all dendrites, axons, glia and synapses, we show that high-end light microscopy is sufficient to study densely labeled populations of synapses in neuropil, as well as determine identities of the pre- and post-synaptic fluorophores tagging each individual synapse. Our study was in part motivated by the recent observation by [28] of individual synaptic puncta with wide-field LM on ultra-thin slices. Our results not only confirm but also substantially extend their experimental findings. Using the “gold standard” of electron microscopy, we demonstrate that the light diffraction limit is not a limitation for the optical observation of densely labeled synapses; but also we show that IDLM, such as in array tomography [28], or the simplest super-resolution technique such as SIM [26], allows one to successfully detect and recognize 95–99% of all synapses, and no existing experimental constraints, such as the plausible photon budgets, background pollution, or realistic synaptic protein concentrations, present obstacles to that end. Although our study was performed using a sample from rodent neuropil, its results primarily depend on two parameters: the mean density of synapses in neuropil, ∼1–2 µm^−3^, and the typical size of synapses, ∼300 nm [32], [33], [34], [35], [36], [38]. Our conclusions, therefore, can be generalized immediately to other animals where these parameters are known to be similar.

Based on these findings, we propose a new approach for reconstructing neural connectivity optically, by tagging synapses with arrays of spectrally distinct fluorescent markers, expressed in different combinations in different neurons using Cre/Lox system Brainbow (i.e., synaptic Brainbow) or libraries of genetic promoters. By localizing fluorescent synaptic puncta optically, and identifying the patterns of pre- and post-synaptic fluorophores at different synapses, one can determine the pre- and post-synaptic cells for each synaptic connection, and, thus, reconstruct the connectivity matrix without tracing neural projections – a task presenting formidable challenge both for conventional serial EM and Brainbow LM. Spatial distribution and densities of the synapses of different neurons also can be extracted, although it will not be possible to get the shapes of the dendrites and axons, e.g., necessary to study synaptic inputs integration and similar questions. Our results also describe how well synapses can be detected with fluorescent markers of different wavelengths, given different limiting resolutions with which such puncta can be observed, relevant, e.g., for multi-color arrays for the synaptic Brainbow (Figure 3 and 7). Of course, more accurate bounds also can be obtained for specific arrays of specified fluorochromes using the analytical methods described in this paper.

Because synaptic Brainbow will label only pre- and post-synaptic sites, cell bodies will remain unlabeled. In order to attribute specific synapses to particular neurons in the brain, synaptic Brainbow can be modified slightly. E.g., we may put into the genetic construct a way for the synaptic markers to always co-express together with related fluorescent proteins localized in cell nuclei. This may be achieved, e.g., by placing two coding sequences inside the same loxP bracket in the Brainbow construct, one for the synaptic marker and one for the nuclei-bound protein, or by making expression of the synaptic marker trigger the expression of the respective nuclei-bound protein, etc. In this way, the color-code of each neuron can be read out by looking at its nucleus, and the synapses of that neuron can be found by comparing that color-code with the patterns of synaptic markers found at different puncta.

The main problem of the synaptic Brainbow at this time is the large number of fluorescent markers needed to map a large neural circuit. If *N_c_* is the number of available fluorophores, then the identities of 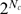 possible synaptic connections can be encoded. E.g., if we multiplex 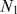 fluorophores on the pre-synaptic side of synaptic clefts, and 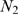 fluorophores on the post-synaptic side (i.e., in total 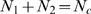 fluorophores), we can distinctly label synapses between any one of 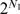 pre-synaptic and 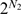 post-synaptic neurons, i.e., 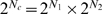 distinct synaptic connections. For a circuit with *N* neurons, the number of connections to be distinguished is *N* ^2^; thus, the smallest number of necessary fluorophores is 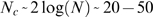, for *N*∼10^4^–10^8^. This should not be viewed as a fatal flaw, however. In fact, since the number of the fluorophores needed to map a circuit here grows only *logarithmically*, described approach currently is the only method with at least theoretical capacity to recover circuits as complex as the entire human brain with *N*∼10^11^ neurons.

One may also consider labeling schemes where the same fluorophore can be used both to label the pre-synaptic sites and the post-synaptic sites. In this way, one may hope to label a greater number of connections with the same number of fluorophores, e.g., 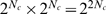. However, complications arise with such schemes, where co-labeling of the pre- and post-synaptic sites with a same color marker can yield a unicolor puncta, or labeling of the pre- and post-synaptic sites with two fluorophores can be confused with the labeling of the post- and pre-synaptic sites with the same colors. Although codes can be designed to avoid such mistakes, the final capacity of any such code will not be greater than 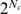. Therefore, we suggest that the synaptic Brainbow should be used with the pre- and post-synaptic markers always distinct.

Certain techniques may be devised to increase the capacity of synaptic Brainbow. E.g., one may take advantage of the continuity of the color of synaptic puncta formed by a neuron, and “trace” the same “color” pre- and post-synaptic puncta through the neuropil. Calculations indicate that *N_c_*≈20 fluorophores will suffice in that case to map local connectivity in an entire cortical column. However, since long range axons may traverse large distances of neuropil without making any synapses, the long range connectivity cannot be mapped in that way.

Another suggestion is to capitalize on possible differences in the expression levels of synaptic markers in different neurons. E.g., in Brainbow mice a limited number of spectrally distinct fluorophores, co-expressed in neurons combinatorially at different levels, generates a much larger number of colors [3], [21]. Thus, an approach is tempting where the expression levels of different synaptic fluorophores can be measured and used to identify neurons. While everywhere in this paper we spoke only of determining whether a fluorophore was or was not expressed, this alternative approach would allow mapping larger neural circuits with fewer spectrally distinct fluorophores.

Unfortunately, as we discussed in Section 3.2., at feasible densities of the synaptic proteins [34], [35], [37] and typical sizes of the synapses [32], [33], most synapses will bind only very small number of fluorophore molecules, ∼10–100 molecules. Since binding of the fluorophore molecules is a random process with certain noise, described by the Poisson statistics, this leads to that the differences in the fluorophore expression levels (between different neurons) will be significantly overshadowed by random fluctuations in the counts of the fluorophore molecules (at different synapses of the same neuron). Therefore, our results indicate that the measurements of the fluorophore expression levels on synapses cannot be done with the accuracy sufficient to identify the host neurons for all but the largest synapses, unlike in the Brainbow mouse. It may still be possible to use this strategy for certain purposes, such as reconstructing the connectivity backbone made of larger synapses, etc.

In our opinion, the most promising approach for using synaptic Brainbow at current time is to combine it with the method such as in [31] for assembling connectivity matrix from multiple animals, which may allow reconstructing the connectivity matrix statistically, using a smaller number of fluorophores but imaging many animals. E.g., mapping of the neural circuit in the entire *Drosophila* brain may be accomplished in this way with *N_c_* = 10–20 fluorophores and imaging of 1000 animals, which is within the capabilities of the existing technology (see [31] for more details).

Another promising approach is to use the results of this paper for studies of the synaptic connectivity at the level of neural populations. By expressing a pre-synaptic marker in one class of neurons, using a genetic promoter, and a post-synaptic marker in another class of neurons, and then collecting labeled synaptic puncta with the methods described here, the connectivity between different classes of neurons may be studied directly, over macroscopically large regions of brain, using optical tools. By multiplexing fluorescent markers, circuits involving ≈100–1000 neural populations (*N_c_*≈10–20) may be mapped efficiently. Libraries of genetic lines, currently under development in several labs, can be used to provide coverage for such a whole brain, neural-class connectivity maps, that would be of great interest to neuroscience.
